# Improvement of psoriasis with ruxolitinib in a patient with myeloproliferative neoplasm: a therapeutic observation^؆^

**DOI:** 10.1016/j.abd.2026.501319

**Published:** 2026-03-23

**Authors:** Luciana Vilela Gomide, Ana Maria Quinteiro Ribeiro

**Affiliations:** Department of Tropical Medicine and Dermatology, Institute of Tropical Pathology and Public Health, Universidade Federal de Goiás, Goiânia, GO, Brazil

Dear Editor,

Psoriasis is a chronic immune-mediated inflammatory skin disease, characterized by persistent inflammation and keratinocyte hyperproliferation. Its pathogenesis involves a dynamic crosstalk among keratinocytes, dendritic cells, and T-lymphocytes.^1^ Recent advances in the understanding of its molecular and immunologic mechanisms have revealed a complex network of cell surface receptors and intracellular signaling pathways that represent promising therapeutic targets.^2^

Among these, Janus Kinase (JAK) inhibitors represent a novel class of therapies capable of modulating key cytokine signaling pathways involved in psoriatic inflammation. While selective Tyrosine Kinase-2 (TYK2) inhibitors, such as deucravacitinib, have recently been approved for moderate-to-severe psoriasis, broader JAK1/2 inhibitors, including ruxolitinib, have not been widely adopted in dermatologic practice due to safety concerns and limited supporting evidence in this indication.^2^, ^3^, ^4^ However, its topical formulation has demonstrated benefit in other inflammatory dermatoses, including atopic dermatitis and vitiligo.^2^, ^3^, ^5^ Here, we report a case in which oral ruxolitinib, prescribed for a hematologic malignancy, resulted in remission of psoriatic skin lesions.

A 63-year-old male with a 20-year history of plaque psoriasis, previously managed exclusively with topical agents, was referred to dermatology in January 2024. He presented with active lesions on the scalp, elbows, periumbilical region, and lower legs, with a Psoriasis Area and Severity Index (PASI) estimated at 14 (Fig. 1).

**Fig. 1:**
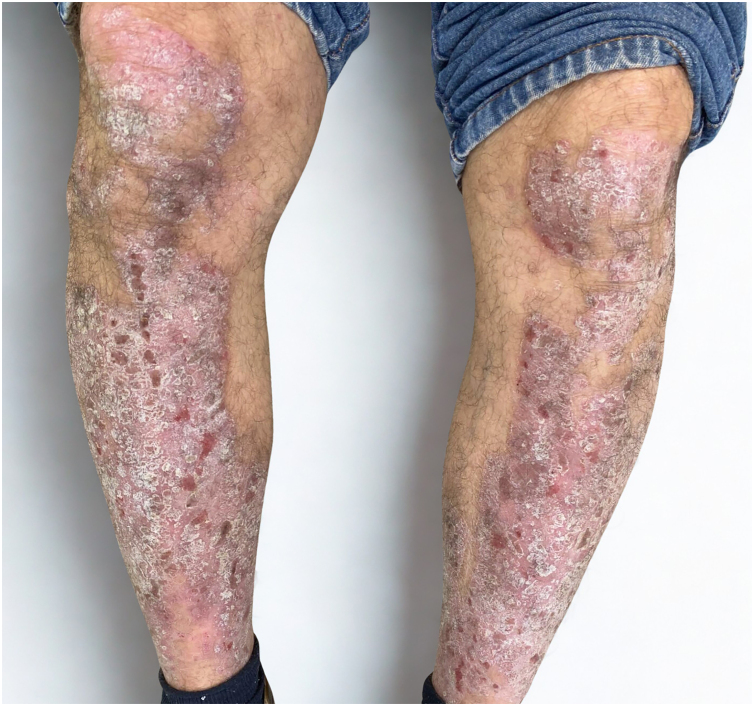
Clinical presentation before ruxolitinib initiation. Extensive erythematous plaques with thick scaling on the lower extremities. Lesions were symmetrical, infiltrated, and associated with marked desquamation (PASI 14).

The patient was diagnosed with primary myelofibrosis in May 2023, classified as Dynamic International Prognostic Scoring System (DIPSS) intermediate-1. Initial treatment with hydroxyurea yielded only a partial hematologic response. On May 2024, ruxolitinib (Jakavi®) was initiated by the hematology team at a dose of 15 mg twice daily. Following initiation, the patient showed progressive and marked improvement of psoriatic lesions (Fig. 2). By November 2024, his PASI had decreased from 14 to 0 (Fig. 3). On follow-up in july 2025, he remained in complete remission.

**Fig. 2:**
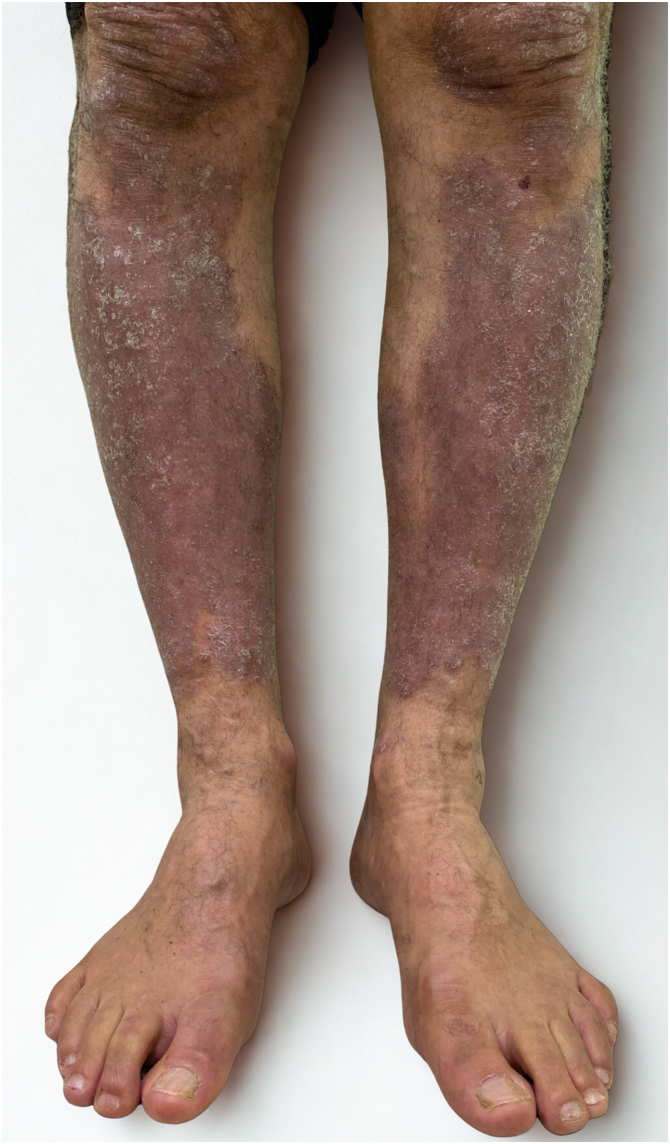
Partial clinical improvement after 30-days of therapy. Noticeable reduction in erythema, infiltration, and scaling of psoriatic lesions on the lower legs.

**Fig. 3:**
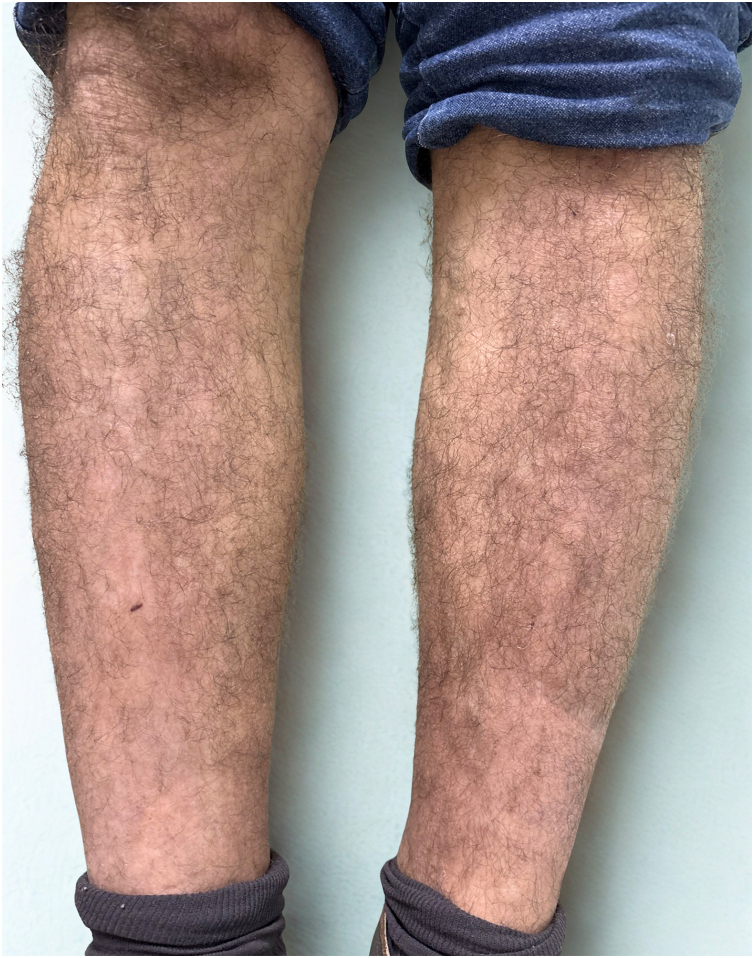
Complete clinical remission after 6-months of therapy, with residual post-inflammatory pigmentation. Patient achieved and maintained PASI 0 without the need for topical or systemic rescue therapy.

Although oral ruxolitinib is not approved for the treatment of psoriasis and carries well-recognized safety concerns, its use in this patient for a concomitant hematologic condition led to complete and sustained remission of cutaneous lesions. This outcome underscores its potential immunomodulatory effect on inflammatory pathways central to psoriatic disease. Among these, the IL-23/IL-17 axis plays a pivotal role in pathogenesis. IL-23, primarily produced by dendritic cells, promotes Th17 differentiation through JAK2 and TYK2 signaling. TYK2, in combination with JAK1 or JAK2, mediates downstream activation of IL-12, IL-23, and type I interferons through STAT3, STAT4, STAT1, and STAT2, supporting the activation and persistence of Th1 and Th17 cells. These effector T-cells, in turn, release proinflammatory cytokines, such as IL-17, TNF-α, IL-22, and IL-26, that drive keratinocyte proliferation and sustain chronic cutaneous inflammation.^2^, ^4^, ^5^, ^6^, ^7^

The therapeutic response observed in this case may be attributed to ruxolitinib’s ability to disrupt these immune signaling pathways via JAK1/2 inhibition. By modulating the downstream effects of key cytokines involved in the psoriatic axis, ruxolitinib may attenuate both Th17 and Th1-mediated inflammation.^2^, ^3^, ^4^, ^5^, ^6^ A similar observation was previously reported in a patient with plaque psoriasis and myelofibrosis secondary to polycythemia vera, who experienced marked cutaneous improvement while receiving oral ruxolitinib for hematologic control.^8^

Ruxolitinib, approved for the treatment of myeloproliferative neoplasms and steroid-refractory graft-versus-host disease, is primarily metabolized via the hepatic CYP3A4 system, with active metabolites eliminated renally. Common non-hematologic adverse events include peripheral edema and diarrhea, while infectious complications such as pneumonia and viral reactivation have been reported. Due to its immunosuppressive and myelosuppressive effects, it may cause anemia, leukopenia, and thrombocytopenia.^9^ In our patient, it was well tolerated, with only mild anemia and no significant complications during follow-up.

Given the safety profile of systemic JAK1/2 inhibition, the use of ruxolitinib in psoriasis may be best reserved for selected cases in which the drug is already indicated for a concomitant condition, as in the present scenario. In such contexts, the dermatologic response may represent a valuable secondary benefit, while therapeutic risk remains justified by the primary hematologic indication.^2^, ^3^, ^5^, ^6^ Although our observation supports the potential efficacy of systemic ruxolitinib in psoriasis, further studies are warranted to better define the role of both systemic and topical JAK inhibitors in the management of psoriatic disease.

## ORCID ID

Luciana Vilela Gomide: 0009-0001-5465-5462

Ana Maria Quinteiro Ribeiro: 0000-0001-9872-0476

## Financial support

This research received no specific grant from any funding agency in the public, commercial or not-for-profit sectors.

## Authors' contributions

Luciana Vilela Gomide: Study conception and planning, preparation and writing of the manuscript; manuscript critical review; intellectual participation in propaedeutic and/or therapeutic management of studied cases; critical literature review; approval of the final version of the manuscript.

Ana Maria Quinteiro Ribeiro: Study conception and planning; manuscript critical review; intellectual participation in propaedeutic and/or therapeutic management of studied cases; approval of the final version of the manuscript.

## Research data availability

Does not apply.

## Conflicts of interest

None declared.
